# Modified T-plate internal fixation in the treatment of medial malleolus fractures of the distal tibia in the elderly

**DOI:** 10.3389/fsurg.2025.1581909

**Published:** 2025-06-16

**Authors:** Xipeng Wang, Kun Nie, Jiangtao Liu, Cong Jiang

**Affiliations:** Department of Orthopaedic Surgery, The Central Hospital of Wuhan, Tongji Medical College, Huazhong University of Science and Technology, Wuhan, China

**Keywords:** tibia fracture, modified T-plate, internal fixation surgery, long-term rehabilitation, cannulated lag screws

## Abstract

**Objective:**

This study aimed to compare the clinical efficacy of modified T-plate internal fixation vs. conventional cannulated lag screws in treating medial malleolus fractures of the distal tibia in elderly patients, with a focus on surgical outcomes, functional recovery, and complication profiles.

**Methods:**

A prospective cohort analysis was conducted on a sample of 46 elderly patients (aged ≥ 60 years) with isolated medial malleolus fractures treated at a single orthopedic center between April 2020 and December 2022. Patients were allocated to either modified T-plate internal fixation (*n* = 23) or cannulated lag screw internal fixation (*n* = 23). The postoperative conditions, including operative time, blood loss, and complications, as well as the long-term rehabilitation outcomes, such as AOFAS ankle and hindfoot score at 3, 6, and 12 months, were systematically compared.

**Results:**

The modified T-plate group exhibited significantly superior early functional recovery, with higher AOFAS ankle and hindfoot score at 3 months (63.5 ± 8.0 vs. 55.3 ± 13.3, *P* = 0.015) and 6 months (74.6 ± 8.9 vs. 67.8 ± 12.5, *P* = 0.041), though operative time was longer (85.6 ± 12.3 vs. 72.4 ± 10.8 min, *P* < 0.001). No significant differences were observed in intraoperative blood loss (120.5 ± 25.6 vs. 115.8 ± 22.4 ml, *P* = 0.511), overall complication rates (8.7% vs. 17.4%, *P* = 0.381), or long-term outcomes of AOFAS ankle and hindfoot score at 12 month (89.0 ± 8.7 vs. 87.9 ± 7.6, *P* = 0.628). Both groups demonstrated comparable safety profiles, with no severe complications during a mean 14.4-month follow-up.

**Conclusion:**

Modified T-plate fixation has been shown to facilitate early functional rehabilitation in elderly patients with distal tibial medial malleolus fractures. Although this method requires marginally longer operative time, it offers equivalent long-term outcomes and safety to traditional lag screws internal fixation. Notably, it is particularly advantageous for osteoporotic patients, as it addresses the challenges posed by bone fragility and compromised healing capacity.

## 1 Introduction

Fractures near the ankle of the distal tibia are frequently encountered in clinical settings, accounting for approximately 10% of all tibial fractures (1, 2). Given that the distal tibia is in close proximity to the ankle joint, it tends to pose significant difficulties in achieving fracture reduction and fixation. Coupled with the poor local soft tissue coverage and blood supply (3), it has emerged as a particularly thorny traumatic fracture type at present.

The treatment modalities for fractures near the ankle of the distal tibia encompass conservative treatment and surgical treatment (4, 5). Conservative treatment comprises skin traction, skeletal traction, plaster or splint fixation, and brace fixation, among others; surgical treatment is generally carried out based on the AO classification of soft tissue injuries, the AO classification of fractures, or the Gustilo classification of open fractures to perform external fixation or internal fixation (6–8).

In clinical practice, method of cannulated lag screws was commonly applied as an internal fixation surgery (9–13). However, these fixation methods all come with certain limitations. This is especially true for elderly patients who often concurrently have varying degrees of osteoporosis. When using cannulated lag screws to fix medial malleolus fractures, factors such as osteoporosis can undermine the stability of screw fixation, necessitating multiple and repetitive use of Kirschner wires during the operation (14, 15), which in turn leads to poor local blood supply. Moreover, following distal tibia fractures, the soft tissue in front of the tibia typically sustains varying degrees of damage as well. Consequently, postoperative complications such as wound skin bleeding, necrosis, and infection are prone to occur, and in severe cases, even secondary surgical intervention may be required (16–18).

The surgical technique of modified T-plate internal fixation for medial malleolus fractures of the distal tibia in the elderly presents a novel surgical option. It can safeguard the local soft tissue to the fullest extent, enlarge the contact area between the distal fracture end and the fracture line, mitigate the impact of bone loss, such as osteoporosis at the fracture site, on fracture healing, and reduce the incidence of postoperative infection and nonunion (19–21).

This study combines the injury mechanism and clinical characteristics of fractures near the ankle of the distal tibia, dissects the strengths and weaknesses of existing internal fixation devices, and employs the modified T-plate to fix medial malleolus fractures, thereby establishing a more reliable internal fixation method. Through prospective analysis, we focus on observing the postoperative functional rehabilitation.

## 2 Methods

### 2.1 Research objectives

Inclusion Criteria: (1) Age ≥ 60 years, both male and female, diagnosed with medial malleolus fractures of the distal tibia; (2) Treated with modified T-plate internal fixation or cannulated lag screw internal fixation.

Exclusion criteria: (1) Combined with ankle fractures; (2) Bilateral fractures near the ankle of the distal tibia; (3) Previous history of tibial trauma and surgery; (3) Age < 60 years; (4) Incomplete clinical data or follow-up time < 18 months.

### 2.2 General conditions of patients

A prospective study was conducted on elderly patients with medial malleolus fractures of the distal tibia who underwent modified T-plate internal fixation or cannulated lag screw internal fixation in the Department of Orthopedics, Wuhan Central Hospital, Tongji Medical College, Huazhong University of Science and Technology from April 2020 to December 2022.

Patients were grouped according to Herscovici staging (22), as determined by preoperative imaging. Patients with type B were treated with cannulated lag screw internal fixation, while those with type C or D underwent modified T-plate internal fixation.

### 2.3 Surgical procedure

**Figure 1:**
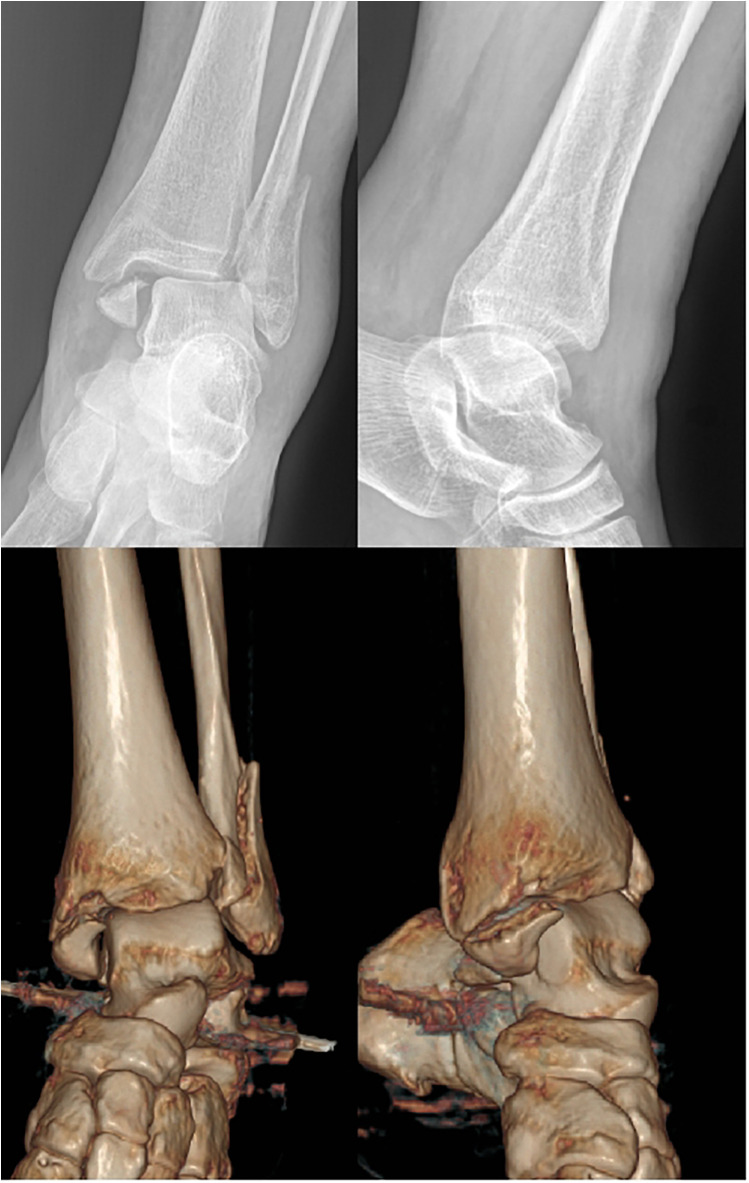
A 46-year-old female patient was admitted to the hospital due to “an accidental fall 3 h prior”. Imaging examinations of the affected limb, consisting of x-ray (upper two images) and 3D CT scan (lower two images) revealed fractures of the left medial and lateral malleoli accompanied by subluxation of the ankle joint.

Before the operation, based on the patient's x-ray films and the three-dimensional CT imaging system showing the medial malleolus fracture situation, a three-dimensional finished product was printed and directly used for internal fixation during the operation (Figure 1) (23, 24).

After the patient received epidural anesthesia or general anesthesia, they were placed in a supine position. An air tourniquet was placed at the root of the affected thigh, and the pressure was set at 45–60 kPa. The surgical area was disinfected and draped following the conventional procedures, and the fracture reduction was assisted by a “C”-arm x-ray machine. An approximately 3-cm-long incision was made along the tip of the medial malleolus, and the soft tissue at the fracture end was carefully removed. The reduction forceps and Kirschner wires were used to complete the reduction and provide temporary fixation.

After the fracture reduction was finished, a 3-hole or 5-hole plate bender was selected to reshape the neck of the T-plate, and the bending angle was selected to be approximately 50°–65°. After the plate was well-shaped, the screw holes were placed in the direction opposite to that of the Kirschner wires, and then the screws were inserted. Alternatively, cannulated lag screw(s) was driven directly to stabilize the fractures.

**Figure 2:**
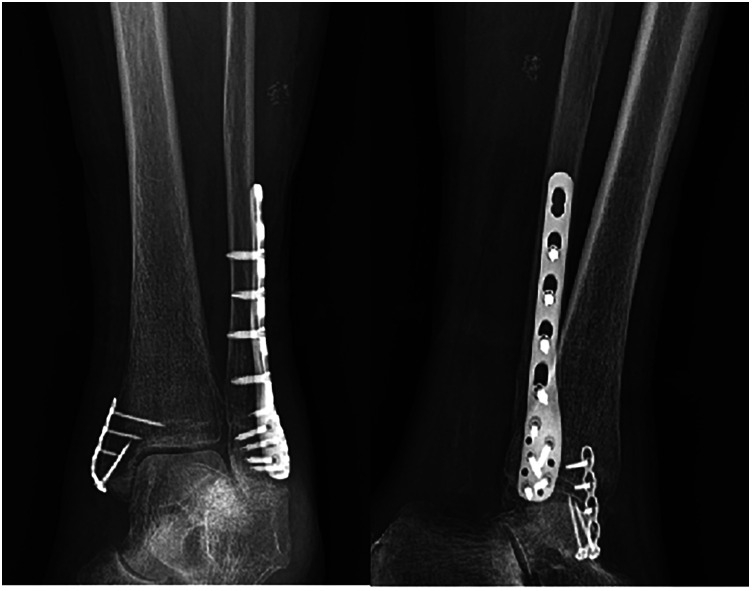
After exclusion of surgical contraindications, the patient underwent open reduction and internal fixation (ORIF) for the ankle fracture, with the medial malleolus fracture treated using mini-plate internal fixation.

At the end of the operation, the affected ankle could be held and moderately rotated to observe whether there was any abnormal movement at the fracture end to judge the stability of the reduction. Finally, the “C”-arm x-ray machine was used again for fluoroscopy to confirm the actual situation of the fracture reduction and fixation (Figure 2). All surgeries are performed by a team with the same lead surgeon to minimize possible bias.

### 2.4 Postoperative management

Postoperatively, non-steroidal anti-inflammatory drugs were administered for analgesic treatment. Whether to place a drainage tube was determined based on the wound condition. The drainage tube was removed 24–48 h after the operation or when the drainage volume was less than 20 ml. Broad-spectrum antibiotics were routinely used to prevent infection, and routine anticoagulation was carried out to prevent deep vein thrombosis of the lower extremities.

The two groups adopted the same postoperative rehabilitation protocol. Twenty-four hours after the operation, patients were encouraged to perform straight leg raises and ankle pump exercises to promote blood circulation. For straight leg raises, patients should first extend the knee joint and then slowly lift the affected limb to about 45°. They were required to complete 10–20 sets per day, with 5–10 repetitions per set. For ankle pump exercises, patients were asked to dorsiflex the ankle forcefully and then plantarflex it to the maximum range. They needed to complete 20–30 sets per day, with 10–20 repetitions per set.

### 2.5 Follow-up and observation indicators

Patients were followed up in the outpatient clinic at 6 weeks, 3, 6, 12, and 18 months after the operation. The operation time, intraoperative blood loss, hospital stay, time to full weight-bearing, and fracture healing status were recorded. The American Orthopaedic Foot and Ankle Society (AOFAS) ankle and hindfoot score (25) were used to evaluate the recovery of patients' living and athletic abilities.

The AOFAS ankle and hindfoot score includes the evaluation of pain, walking function, and alignment of the foot and ankle, with a full score of 100 points. Among them, 90–100 points are rated as excellent, 80–89 points as good, 70–79 points as acceptable, and <70 points as poor.

### 2.6 Ethical approval

This study was approved by the Ethics Committee of the Central Hospital of Wuhan (Approval No.WHZXKYL2025-076). Written informed consent was obtained from all participants after explaining potential risks and benefits. Data were stored in a password-protected database with access limited to our research team.

### 2.7 Statistical analysis

Statistical analysis was performed using SPSS 22.0 statistical software (IBM, USA). The measurement data that passed the normality test and homogeneity of variance were represented by mean ± Std., and the ANOVA was used for comparison. The count data were represented by frequency (cases), and the Chi-square test was used for comparison. The significance level α was set at 0.05 for two-sided tests.

## 3 Results

### 3.1 General conditions of patients

There were totally 46 cases, including 26 males and 20 females, with an average age of 66.65 ± 5.22 years (range: 60–83 years). 23 cases was underwent modified T-plate internal fixation and another 23 cases was underwent cannulated lag screw internal fixation (Table 1). And these cases showed no significant difference between modified T-plate group and cannulated lag screw group (*P* < 0.05).

**Table 1:** Comparison of general conditions of patients.

Comparison	Modified T-plate	Cannulated lag screw	Statistic	*P* value
Age [year(s)]	67.65 ± 5.36	65.65 ± 5.00	t = 1.31	0.197
Gender (male/female)	16/7	10/13	*χ*^2^ = 3.19	0.136

### 3.2 Perioperative conditions of patients

All patients successfully completed the surgery. As shown in Table 2, the operating time of the modified T-plate group was averagely 13 min longer than that of the cannulated lag screw group, with the former being 85.6 ± 12.3 min and the latter being 72.4 ± 10.8 min (*P* < 0.001). And there was no statistically significant difference in intraoperative blood loss (*P* = 0.511) or complication happen (*P* = 0.381) between them. Specifically, there was no significant difference in the types of complications between them, including incision infection, internal fixation bleeding, and delayed fracture healing (*P* = 0.694).

**Table 2:** Comparison of perioperative conditions of patients.

Comparison	Modified T-plate	Cannulated lag screw	Statistic	*P* value
Operative time [minute(s)]	85.6 ± 12.3	72.4 ± 10.8	*t* = 3.87	<0.001
Intraoperative blood loss (ml)	120.5 ± 25.6	115.8 ± 22.4	*t* = 0.66	0.511
Complication (%)	8.7%	17.4%	*χ*^2^ = 0.767	0.381
Incision infection [case(s)]	1	1	*χ*^2^ = 0.737	0.694
Internal fixation loosening [case(s)]	1	2		
Delayed fracture healing [case(s)]	0	1		

### 3.3 Long-term rehabilitation of patients

As shown in Table 3, Modified T-plate group demonstrated a statistically significant advantage over cannulated lag screw group in early recovery of 3 months postoperative in AOFAS ankle and hindfoot score (*P* = 0.015). This advantage was maintained until 6 months postoperative, but the gap has decreased (*P* = 0.041). And by 1 year postoperative, the scores of both are similar, with no significant difference (*P* = 0.628).

**Table 3:** Comparison of long-term rehabilitation of patients.

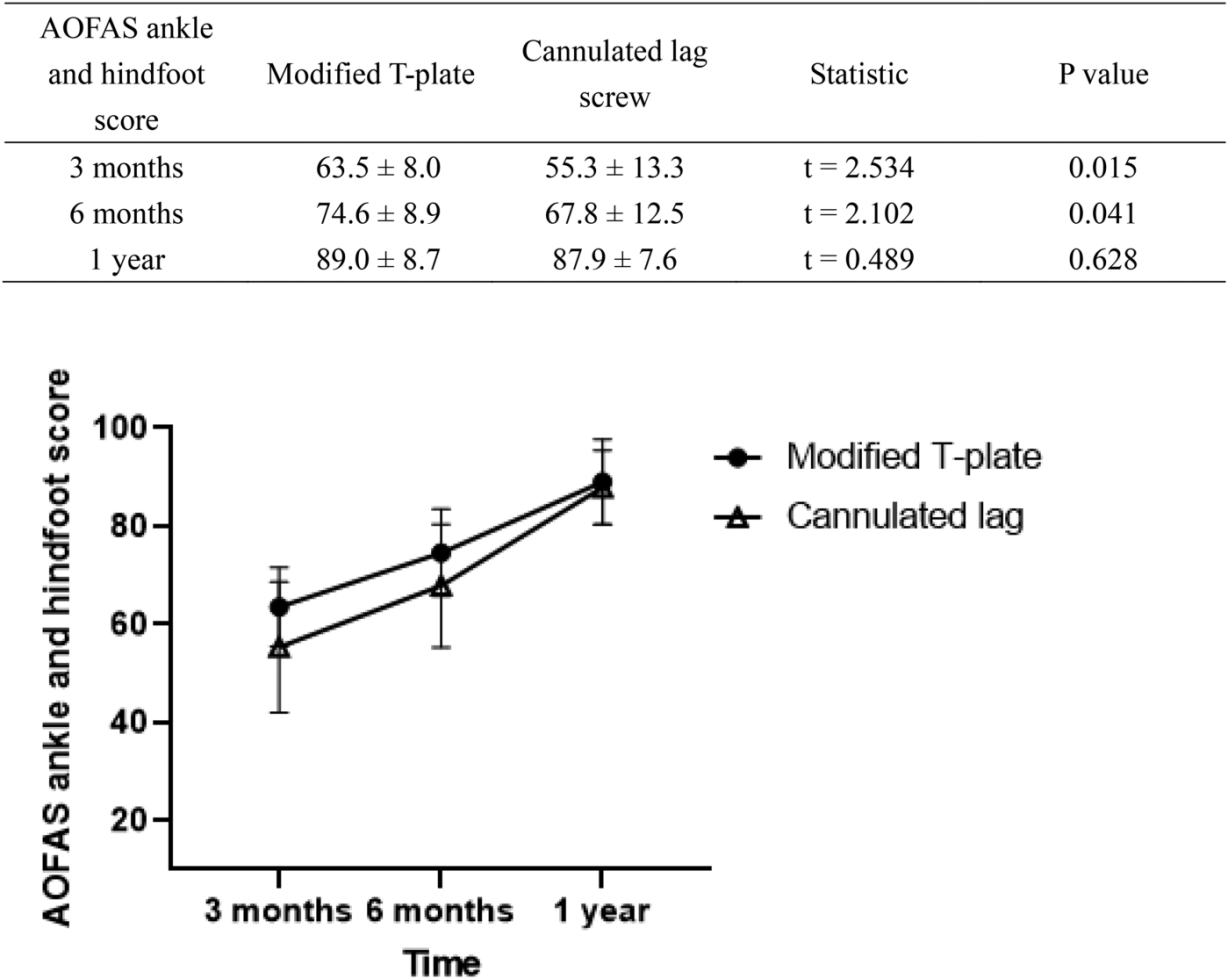

### 3.4 Postoperative complications

All patients were followed up, and the follow-up period was (14.4 ± 3.5) months (range: 6–18 months). At the last follow-up, none of the patients had complications such as ankle joint stiffness, neurovascular injury, deep vein thrombosis of the lower extremities, internal fixation infection or breakage.

## 4 Discussion

### 4.1 Anatomical characteristics of distal tibial medial malleolus

Typically, the region 10–11 cm distal to the supra-ankle part of the tibia is defined as the distal tibia near the ankle. The medial malleolus is mainly composed of thick cortical bone, and as it extends downward to the tip of the medial malleolus, it gradually transitions into cancellous bone with a thin cortex (2, 26). This part of the tibia forms an inverted trumpet-shaped medullary cavity that is narrow at the top and wide at the bottom, mainly on the medial side.

### 4.2 Indications of modified T-plate internal fixation

For medial malleolus fractures of the distal tibia, it encompasses both closed and open varieties, irrespective of whether the fracture is displaced or not. According to the AO classification, subtypes like AO/OTA 43.A1, AO/OTA 43.A2, and AO/OTA 43.A3 are involved (27, 28).

In cases of medial malleolus fractures of the distal tibia that involve the ankle joint, such as AO/OTA 43.C1 and AO/OTA 43.C2. It is particularly apt for elderly patients with medial malleolus fractures, whether accompanied by osteoporosis or not. In these geriatric patients, due to advanced age leading to diminished bone quality, the application of modified T-plate internal fixation can offer a more dependable fixation outcome. Firstly, the substantial contact area between the plate and the bone can distribute stress, curtailing the risk of re-displacement at the fracture site. Secondly, for those with osteoporosis, its bespoke design augments the grip on regions with relatively porous bone, promoting fracture healing. Moreover, when confronted with fractures of varying complexity, such as comminuted fractures or those with bone defects, satisfactory reduction and fixation can still be accomplished through judicious shaping and the use of multiple screws, laying a solid foundation for subsequent rehabilitation.

### 4.3 Contraindications of modified T-plate internal fixation

Severe local soft tissue injury or infection: When there is a severe crush injury, a defect in the soft tissue at the fracture site, or conspicuous signs of infection, like an open fracture combined with severe contamination and without effective debridement, the employment of modified T-plate internal fixation is contraindicated. The rationale is that the internal fixation device might serve as an ideal breeding ground for bacteria, rendering the infection arduous to manage and potentially precipitating grave complications like osteomyelitis. For illustration, in the context of a severe open tibial fracture accompanied by extensive soft tissue defects and contamination, debridement and soft tissue repair ought to be executed first, rather than hastening to perform plate internal fixation.

Poor general condition of the patient unable to tolerate surgery: If patients have underlying ailments such as severe cardiopulmonary dysfunction and coagulation disorders and cannot endure the trauma and bleeding associated with surgery, modified T-plate internal fixation is ill-suited. Consider patients with severe coronary heart disease, heart failure, or uncorrected coagulation factor deficiencies. Surgery in such cases might instigate life-threatening scenarios like cardiac arrest and profuse bleeding.

Special anatomical structure at the fracture site not suitable for T-plate: When the anatomical configuration at the fracture site is highly idiosyncratic and the T-plate fails to conform well or efficaciously immobilize the fracture fragments. In instances where the type of medial malleolus fracture does not synchronize with the design of the T-plate, alternative types of internal fixation devices, such as Kirschner wires or intramedullary nails, might need to be opted for.

### 4.4 Advantages of modified T-plate internal fixation

#### 4.4.1 Anatomical reduction and stability

Conforming to Bone Shape: The anatomical structure of the medial malleolus is relatively complex, and the shape of the modified T-plate can well adapt to its morphology. Even in elderly patients with osteoporosis, this closely fitting characteristic still helps to accurately reduce the fracture fragments to the anatomical position. Just like a jigsaw puzzle, the shape of the T-plate can guide the fracture fragments back to the correct position, providing favorable initial conditions for fracture healing.

Multi-directional Fixation: The T-plate has multiple screw holes and can fix the fracture fragments from different directions. For the comminuted fracture situations that may occur in elderly patients after fractures, the advantage of this multi-directional fixation is more obvious. It can act like a firm “grabber”, stably fixing the fracture fragments together, reducing the risk of the fracture fragments moving after surgery, and helping to maintain the stability of the fracture site.

#### 4.4.2 Support for early activity

Providing Certain Support Force: Although elderly patients have osteoporosis, in the initial stage of fractures, the modified T-plate can provide necessary support for the medial malleolus. When patients attempt to perform early non-weight-bearing activities, such as flexion and extension of the ankle joint in bed, the plate can help maintain the stability of the fracture site and prevent the fracture from displacing again. This has a positive significance for reducing the complications caused by long-term bed rest, such as pulmonary infection and deep vein thrombosis.

Promoting Functional Recovery: Early and moderate activities are also important for the joint function recovery of elderly patients. After internal fixation with the modified T-plate, under the guidance of a doctor, patients can gradually carry out rehabilitation training for the ankle joint. This helps to improve the range of motion of the ankle joint, increase muscle strength, avoid joint stiffness and muscle atrophy, and thus improve the self-care ability of elderly patients.

#### 4.4.3 Characteristics of surgical procedure

Mature and Easy-to-Operate Technique: For surgeons, the T-plate internal fixation technique is a relatively mature surgical method. When dealing with medial malleolus fractures in elderly patients, surgeons can perform the surgical operation relatively skillfully. This can reduce the operation time and lower the surgical risk. Especially for elderly patients who may not tolerate long and complex surgical procedures due to their physical conditions, the mature technique can relieve this burden to a certain extent.

Good Visibility: During the operation, the medial malleolus area is relatively easy to expose, and surgeons can clearly see the fracture site and the surrounding anatomical structures when placing the modified T-plate. This favorable visibility helps to ensure the correct placement of the plate and the accurate insertion of screws, improving the accuracy of the operation. Even in the case of relatively fragile bones in elderly patients, it can minimize damage to the surrounding tissues.

In addition, compared with traditional cannulated lag screw internal fixation, for medial malleolus fractures in elderly patients with osteoporosis (29, 30), due to the decreased bone density and sparse trabeculae, the screws of the modified T-plate can obtain sufficient grip. This can prevent the screws from loosening or pulling out after surgery, thus affecting the fixation effect of the fracture and increasing the risk of fracture displacement.

Stress Shielding and Bone Resorption: After the plate is implanted, it will share the stress borne by the bone, generating a stress shielding effect. In elderly patients with osteoporosis, this stress shielding may be more pronounced. The use of modified T-plate internal fixation, as the physiological stress on the bone is reduced, will further cause bone resorption, thus promoting early fracture healing.

### 4.5 Disadvantages of modified T-plate internal fixation

#### 4.5.1 Soft tissue complications

Skin Problems: The skin of elderly patients is usually thin and has poor elasticity. After the modified T-plate is placed around the medial malleolus, it may exert relatively large pressure on the skin, increasing the risk of skin breakdown and necrosis. Especially in the case of postoperative local swelling or poor blood circulation, the friction and pressure between the skin and the plate may trigger skin problems.

Blood Supply Impact: The placement of the plate during the operation may interfere with the blood supply of the surrounding soft tissue. Elderly patients themselves have poor vascular elasticity and compensatory capacity. Once the blood supply is affected, the blood supply to the fracture site will be reduced. This will not only delay the speed of fracture healing but also increase the risk of infection, because good blood supply is an important guarantee for resisting infection.

Longer Operative Time: Due to the secondary contouring of the T-plate during the surgery, the operation often required more time than the cannulated lag screw surgery. This might result in increased intraoperative bleeding and/or the development of postoperative infections. However, the results of our study did not demonstrate such differences.

#### 4.5.2 Later problems

Plate Removal Risks: If plate removal is considered, elderly patients may face risks associated with reoperation. Elderly patients may experience re-fracture during the postoperative recovery period.

Rehabilitation Difficulties (31): The physical function of elderly patients declines, and their metabolism is slow. The process of fracture healing and rehabilitation is relatively long. Even with the assistance of the modified T-plate, the rehabilitation effect may not be as ideal as that of young patients. During the rehabilitation process, they may be more likely to experience various complications, such as slow recovery of joint movement and difficulty in restoring muscle strength, thus affecting the overall treatment effect and quality of life.

### 4.6 Research limitations

This study still has certain limitations: (1) The relatively small number of samples might not fully represent the overall patient population, limiting the generalizability of the findings (32). (2) Currently, the methods used to evaluate long term rehabilitation of patients in clinical practice, including AOFAS ankle and hindfoot scores, are actually weighted semi quantitative scoring scales rather than quantitative measurements, resulting in slightly weaker comparability of data. (3) The follow-up period was relatively short, which may have underestimated the incidence of complications. Longer-term follow-up is necessary to capture potential late-onset complications that could impact patient outcomes. (4) This study did not compare with intramedullary fixation. Without a direct comparison to this alternative fixation method, it is difficult to comprehensively evaluate the relative advantages and disadvantages of the modified T-plate internal fixation, leaving room for further investigation.

## 5 Conclusion

This prospective comparative study demonstrates that modified T-plate internal fixation offers distinct advantages over conventional cannulated lag screws internal fixation for treating distal tibial medial malleolus fractures in elderly patients. Key findings reveal that the modified T-plate internal fixation achieved significantly superior early functional recovery (AOFAS scores: 63.5 vs. 55.3 at 3 months, *P* = 0.015; 74.6 vs. 67.8 at 6 months, *P* = 0.041), attributed to enhanced biomechanical stability and optimized soft tissue preservation. Although its operative time was marginally longer (85.6 vs. 72.4 min, *P* < 0.001). No differences in intraoperative blood loss, complication rates, or long-term outcomes (AOFAS 89.0 vs. 87.9 at 12 months, *P* = 0.628) were observed.

The modified T-plate internal fixation effectively mitigates challenges posed by osteoporosis and fragile soft tissues in elderly populations, promoting early mobilization while maintaining fracture alignment. Limitations of this study include the single-center design and moderate sample size; future multicenter studies with extended follow-ups are warranted to validate these findings. This technique represents a promising advancement in geriatric fracture management, aligning with principles of anatomical restoration and biological fixation.

## Data Availability

The raw data supporting the conclusions of this article will be made available by the authors, without undue reservation.
